# A Systematic Literature Review of Self-Reported Smoking Cessation Counseling by Primary Care Physicians

**DOI:** 10.1371/journal.pone.0168482

**Published:** 2016-12-21

**Authors:** Anna-Lena Bartsch, Martin Härter, Jasmin Niedrich, Anna Levke Brütt, Angela Buchholz

**Affiliations:** Department of Medical Psychology, University Medical Center Hamburg-Eppendorf, Hamburg, Germany; Legacy, Schroeder Institute for Tobacco Research and Policy Studies, UNITED STATES

## Abstract

Tobacco consumption is a risk factor for chronic diseases and worldwide around six million people die from long-term exposure to first- or second-hand smoke annually. One effective approach to tobacco control is smoking cessation counseling by primary care physicians. However, research suggests that smoking cessation counseling is not sufficiently implemented in primary care. In order to understand and address the discrepancy between evidence and practice, an overview of counseling practices is needed. Therefore, the aim of this systematic literature review is to assess the frequency of smoking cessation counseling in primary care. Self-reported counseling behavior by physicians is categorized according to the 5A’s strategy (ask, advise, assess, assist, arrange). An electronic database search was performed in Embase, Medline, PsycINFO, CINAHL and the Cochrane Library and overall, 3491 records were identified. After duplicates were removed, the title and abstracts of 2468 articles were screened for eligibility according to inclusion/exclusion criteria. The remaining 97 full-text articles reporting smoking cessation counseling by primary care physicians were assessed for eligibility. Eligible studies were those that measured physicians’ self-reported smoking cessation counseling activities via questionnaire. Thirty-five articles were included in the final review (1 intervention and 34 cross-sectional studies). On average, behavior corresponding to the 5A’s was reported by 65% of physicians for “Ask”, 63% for “Advise”, 36% for “Assess”, 44% for “Assist”, and 22% of physicians for “Arrange”, although the measurement and reporting of each of these counseling practices varied across studies. Overall, the results indicate that the first strategies (ask, advise) were more frequently reported than the subsequent strategies (assess, assist, arrange). Moreover, there was considerable variation in the items used to assess counseling behaviour and developing a standardized instrument to assess the counseling strategies implemented in primary care would help to identify and address current gaps in practice.

## Introduction

Tobacco consumption is a preventable risk factor for non-communicable diseases such as chronic obstructive pulmonary disease (COPD) and cardiovascular disease. Each year, around six million people die from long-term exposure to first- or second-hand smoke worldwide [1]. Globally, one of the guiding instruments for tobacco control is the World Health Organization Framework Convention on Tobacco Control (WHO FCTC) [2]. The convention gives specific recommendations for a number of different tobacco control strategies that should be implemented, such as developing comprehensive smoking cessation guidelines and introducing warning labels on cigarette packages [2]. One approach to reduce tobacco consumption that is recommended in guidelines for the treatment of tobacco dependence is to offer smoking cessation counseling in the primary care setting [3–5]. Smoking cessation counseling by general practitioners (GP’s) has been shown to increase quit rates [6]. The general practice is an appropriate setting for smoking cessation counseling for a number of reasons [7]. First, GP’s have suitable access to the target group because around 80% of the German population visit their GP at least once per year [8]. Second, regular personal contact builds trust between GP’s and patients and facilitates the provision of quit advice [9]. Third, face-to-face contact allows for the delivery of individual smoking cessation advice [10].

The clinical practice guideline of the US Public Health Service contains a comprehensive approach to smoking cessation counseling in primary care settings, which specifies individual counseling steps such as asking about tobacco consumption and recommending the use of pharmacological aids; the 5A’s strategy [5]. The 5A’s refer to a sequence of 5 different counseling strategies: “Ask” (ask all patients about tobacco use), “Advise” (advise all tobacco users to quit), “Assess” (assess the willingness to quit), “Assist” (assist with quitting) and “Arrange” (arrange follow-up) [5]. Examples of other approaches are the ABC model (*Ask* about and document smoking status, give *brief* advice and encourage the use of *cessation* support) and the recommendation of the American Association of Family Physicians (AAFP; Ask about tobacco use, advise to stop using tobacco products and provide behavioral interventions). We focus on the 5 A’s strategy because it distinguishes between 5 counseling steps and is therefore more inclusive than the ABC model and AAFP recommendation which describe 3 counseling steps [4, 5, 11].

Although smoking cessation counseling is effective and recommended in clinical guidelines, it is not fully implemented in primary care [10, 12]. In order to further understand and address the discrepancy between evidence and practice, an overview of current counseling practices is needed. A systematic literature review has examined the frequency of behavioral counseling by physicians for multiple behaviors (tobacco consumption, physical activity and nutrition) and found that the use of educational materials and referral to smoking cessation courses were frequently reported counseling strategies [13]. For example, educational materials were recommended by 58% of Scottish physicians and 61% of Canadian physicians [14, 15]. However, comparability of quantitative information on smoking cessation counseling practices was limited and it is not clear which counseling steps are implemented in practice. Therefore, the aim of this literature review is to systematically assess physician-reported smoking cessation counseling in primary care by classifying counseling practices according to the 5A’s strategy.

## Methods

### Protocol registration and search strategy

The present literature review was reported according to the PRISMA statement [16]. A protocol for this literature review was not registered. The database search was limited to studies published between 2000 and June 2015 because counseling behavior was categorized according to the 5A’s strategy, which was published by the US Public Health Service in the year 2000 and the literature search was conducted in June 2015. A preliminary search of Medline via OVID served to identify relevant keywords. The core search strategy was developed using the keywords and relevant synonyms to capture smoking cessation counseling by physicians and reviewed by a librarian experienced in database searches. Among the main keywords used in the search were: “smoking cessation”, “counseling” and “primary care physician”. The search strategy was then adapted in order to perform an extensive literature search in the following databases: Embase, Medline and PsycINFO via OVID as well as CINAHL and the Cochrane library (Please see S1 File for the detailed search strategy per database). Further articles were identified by screening the bibliographies of articles retrieved from the initial search.

### Study selection

Two independent reviewers (ALBa and JN) performed the title and abstract screening of the studies identified by the search based on a checklist for the inclusion/exclusion of studies framed in relation to PICOS (Population, intervention, comparator, outcome and study design; see Table 1). The reliability of the checklist was tested on a random selection of 100 articles prior to the title and abstract screening. Studies for which all items on the checklist were answered with “yes” were included in the review. There was substantial agreement (Cohen’s Kappa = 0.66) between the two reviewers (JN and ALBa), who then performed the full-text screening independently. Any discrepancies were resolved by discussion until consensus was reached.

**Table 1:** Inclusion/Exclusion criteria checklist.

Inclusion/Exclusion criteria		Yes	No
**Population**			
**A.**	Study population are primary care physicians (defined as either general practitioners, family physicians, internists)?	□	□
**Intervention**			
**B.**	Study reports on smoking cessation counseling delivered by primary care physicians to patients? Patients receiving the intervention are at least 18 years of age?	□	□
**Comparator**			
**C.**	Primary care physicians provide smoking cessation counseling vs. those that do not provide it?	□	□
**Outcome**			
**D.**	Reports proportions of primary care physicians engaging in smoking cessation counseling?	□	□
**Study design**			
**E.1**	Measure is self-report questionnaire?	□	□
**E.2**	Does not present patient-reported data?	□	□
**E.3**	Reports baseline data from intervention studies?	□	□
**E.4**	Published in English or German language?	□	□
**E.5**	Not dissertation?	□	□
**E.6**	Not conference proceeding?	□	□
**E.7**	Not qualitative study?	□	□
**E.8**	Published manuscript?	□	□
**Inclusion**	All bold fields answered with “yes”		

### Study inclusion/exclusion criteria

Studies were included in the review if they were published in either English or German language and measured physicians’ self-reported smoking cessation counseling activities via questionnaire. The main outcome was the proportion of physicians reporting smoking cessation counseling. We excluded studies that reported the proportion of patients receiving smoking cessation counseling because it has been shown that physician and patient-reported data differ [17]. Research articles that defined the majority of the study population as either general practitioners, family physicians or internists (referred to as physicians in the following) were included in the review [18]. Research has shown that physicians provide smoking cessation counseling to young patients more frequently than to adults. We therefore excluded articles with study populations consisting only of paediatricians or young patients (below age 18) [19].

### Data extraction

A data extraction sheet was pilot-tested and adjusted as necessary in order to obtain relevant data from the articles. One reviewer (ALBa) performed the initial data extraction and a second reviewer (BS) checked whether the data were extracted accurately. The following information was extracted: study identifiers (Author, year, and country), the sample (physician characteristics), the methods (data collection method) and results (response rate, sample characteristics and smoking cessation counseling behavior). The proportion of physicians providing smoking cessation counseling to patients was extracted and categorized according the 5A’s. [5].

### Quality assessment

Quality assessment was conducted using the Mixed Methods Appraisal Tool (MMAT) to assess the quality of various study types (e.g. quantitative studies, mixed methods studies etc.) [20]. The tool’s validity and reliability have been established [21, 22]. An overall quality score is determined using criteria that vary by study design. Quality assessment was performed independently by two reviewers (ALBa and BS) and the quality assessment for each study is shown in Table 2.

**Table 2:** Descriptive data of included articles.

Reference & Country	Sample & Response rate (%)	Gender (%) & Age	Data collection	Overall	Physician-reported counseling (% and items ^a^)					Response
				question^a^	Ask	Advise	Assess	Assist	Arrange	options^b^
Alomari et al. (2013) Jordan [23] ***	N = 124FPsRR: 95	Male:48Age: *M* = 39.5 ± 8.7	Self-report questionnaire, Distribution: in person	Behaviour performed or not?	67, Ask about smoking status	66, Advise patients to quit smoking	40, Assess patient willingness to quit	28, Discuss counseling options	7, Arrange follow-up visits with patients to address smoking	**Yes**; No
Barengo et al. (2005) Finland [24] ****	N = 707 GPs RR: 69	n.r.	Self-report questionnaire, Distribution: via post, Developed/adapted from 1998 WHO-questionnaire	n.r.	n.r.	Cardiovascular:Fem.: 87, Male: 84,, Lung: Fem.: 96, Male: 93, "How many percentages of the patients of following groups receive smoking advice?"	n.r.	n.r.	n.r.	**>70%**; 30–70%; <30%
Boldemann et al. (2006) Sweden [25] ****	N = 621, GPs, RR: 64	Male: 55, Age: n.r.	Self-report questionnaire, Distribution: via post	"How many patients who wanted to quit smoking were offered each of the following types of support during the previous month?"	n.r.	61, Advise Nicotine Replacement Therapy	45, Discuss a quit date	25, Provide self-help material	34, Offer individuals smoking cessation follow-up at the clinic	11 or more; 6–10; **1–5**; no patient
Crawford et al. (2005) US [26] **	N = 215, FPs, internists, RR: 24	n.r.	Self-report questionnaire, Distribution: via fax, Developed through literature review & expert consultation, 15 items	n.r.	67, Always ask patients if they use tobacco	81, Advise quitting	45, Always assess tobacco users' willingness to quit	45, Always assist tobacco users with quit attempts	41, Always arrange follow-up for patients willing to quit	n.r.
Curd et al. (2004) US [17] *	N = 241, GPs, FPs, internists, RR: n.r.	Male: 69, Majority (68) ≥ 40 years	Self-report questionnaire, Distribution: via post, in person, Developed by the researchers	"…how often they provided counselingon the topics"	n.r.	51, Topic: "Smoking cesssation in smokers"	n.r.	n.r.	n.r.	**≥75%**; 50–74%; 25–49%; <25%
Ganry & Boche (2004) France [27] ***	N = 503, GPs, RR: 31	Male: 77, Age *M* = 47 ± 6	Self-report questionnaire, Distribution: via post, Developed without pre-existing model, 71 items	"We asked the GPs whether they systematically questioned their patients about their smoking habits."	Fem.: 98, Male: 99, "Do you ask about smoking habits?"	Fem.: 59, Male: 48, "Do you perform smoking cessation yourself?"	n.r.	Fem.: 20, Male: 28, "Do you propose posters and brochures concerning the risks of smoking?"	n.r.	**Yes**; No
Gokirmak et al. (2010) Turkey [28] ***	N = 158, GPs, RR: 93	Male: 81, Age: *M* = 34.9 ± 5.7	Self-report questionnaire, Distribution: n.r., Developed/adapted from 2005 WHO-questionnaire, 45 items	n.r.	47 Frequency of asking patients about their smoking status during the month preceding the filling up of the questionnaire	14 Average number of patients whom they advised to quit smoking during the month preceding the filling up of the questionnaire	n.r.	n.r.	n.r.	Ask**: Frequently**; Sometimes; Don't ask, Advise: **More than 30;** 11–30; 1–10; no patients
Gottlieb et al. (2001) US [29] ***	N = 110, FPs, RR: 93	n.r.	Self-report questionnaire, Distribution: in person	"How often do you …"	59, "How often do you ask the patient about tobacco use?"	81, "How often do you advise the patient to quit smoking?"	22, "How often do you advise setting a specific quit date?"	2, "How often do you refer the patient to a group clinic or intensive smoking cessation program?"	n.r.	**Always**; **Usually**; Often; Half the Time; Sometimes; Rarely; Never
Helgason & Lund (2002) Sweden, Norway, Finland, Iceland [30] ****	N = 2139, GPs, RR: 67	Male: 65, Age: n.r.	Self-report questionnaire, Distribution: via post, Developed based on in-depth interviews, pilot-tested		n.r.	n.r.	13, "Discuss a quit date with the patient"	54, "Advice nicotine replacement therapy"	17, "Offer individual follow-up at the clinic"	**Yes in approxamately ___% of cases**; No never
Hu et al. (2003) US [31] **	N = 406, GPs, FPs, internists, RR: 21	Male: 71, Age: Majority (35) between 40–49	Self-report questionnaire, Distribution: via post, Developed based on expert reviews, pilot-tested, 43 items	"How often during the past 30 days they …"	49, "… asked their patients about tobacco use as part of their examination or history taking"	55, "… advised the patient to quit when they saw a patient who used tobacco"	11, "… advised a smoker who was willing to quit to set a specific quit date"	16, "… prepared patients for withdrawal symptoms"	3, "… had a staff member schedule the patient for follow-up contact,… after setting of a quit date"	**Always**; More than half the time; About half the time; Rarely; Never
Hung et al. (2014) US [32] ***	N = 497, Majority PCPs, RR: 79	Male: 38, Age: *M* = 42.3 ± 10.5	Self-report questionnaire, Distribution: via internet, post	n.r.	82, "During the past month, for how many new patients on a first visit did you ask about tobacco use status?"	61, "For how many of your patients who are tobacco users did you: give advice or counsel to quit?"	36, "For how many of your patients who are tobacco users did you: assess the patient’s readiness to quit?"	28, "For how many tobacco users who were ready to quit did you assist by prescribing nicotine replacement therapy or bupropion?"	13, "For how many tobacco users who were ready to quit did you arrange follow-up for tobacco use in person or by phone?"	**All/most patients**; Many; Half; Few; None
Invernizzi et al. (2001) Italy [33] ***	N = 428, FPs, RR: 67	Male: 84, Age: *M* = 45	Self-report questionnaire, Distribution: in person	n.r.	46, "Counsels adults against smoking"	n.r.	n.r.	n.r.	n.r.	n.r.
Josseran et al. (2000) France [34] ****	N = 2073, GPs, RR: 67	Male: 79, Age: (m) *M* = 46 ± 7.4, (f) *M* = 41.5 ± 7.4	Self-report questionnaire, Distribution: via telephone, Pilot-tested	n.r.	60, Attended to 1 or more smoking patients in the last 7 days	n.r.	n.r.	66, "Two thirds of physicians recommended NRT to their patients"	n.r.	**1 or more patient**; 0 patients
Katz et al. (2012) Canada [35] **	N = 1720, FPs, RR: 43	Male: 45, Age: *M* = 43.6	Self-report questionnaire, Distribution: via post, Developed by experts in survey methodology, pilot-tested	n.r.	79, "I ask patients at their annual visits if they use tobacco"	n.r.	n.r.	14, "I prescribe nicotine replacement to my patients to assist with smoking cessation"	5, "…, how often do you follow up with patients?"	**Always**; Often
Kim et al. (2004) Korea [36] ****	N = 1247, PCPs, RR: 69	n.r., Age: *M* = 42.6 ± 5.8	Self-report questionnaire, Distribution: via post, Adapted from 2002 WHO questionnaire, reviewed by experts, pilot-tested	"They rated how often they …"	60, "… asked clients about their smoking status"	77, "… advised clients to quit smoking"	55, "… assessed clients’ willingness to make an attempt to quit"	21, "… assisted clients who wanted to stop smoking by providing referrals and advice"	11, "… arranged a follow-up visit or phone call to discuss quitting"	**Always**; **Very often**; Often; Occassionally; Never
Kössler et al. (2002) Austria [37] ****	N = 1395, GPs, internists, RR: 18	n.r.	Self-report questionnaire, Distribution: via post, telephone, Developed by the researchers	n.r.	GPs: 50, Internists: 100, "Do you ask patients whether he or she smokes?"	GPs: 85, Internists: 92, "Do you advise every smoker to stop smoking?"	n.r.	n.r.	n.r.	**Yes**; No
Kruger et al. (2015) US [38] ***	N = 2204, FPs, GPs, internists, RR: 83	Male: 71, Age: Majority (43 between 36–45)	Self-report questionnaire, Distribution: via internet	"For tobacco users who visited you over the last year, did you consistently…"	97, "… ask and document whether they use tobacco?"	99, "… recommend they quit using tobacco?"	n.r.	98, "… try to help them quit tobacco by doing any of the following (3 options)?"	48, "… schedule a follow-up visit to help them quit tobacco?"	**Yes**; No
Longo et al. (2006) US [39] ***	N = 346, FPs, internists, RR: 56	Male: 73, Age: *M* = 44	Self-report questionnaire, Distribution: via post, Developed by researchers and physicians, pilot-tested	n.r.	49, "I screen established patients for tobacco use"	63, "To what extend do you urge your patients who smoke to quit?"	38, "To what extent do you ask every patient who smokes if he or she is willing to make a quit attempt at this time?"	n.r.	n.r.	**Always do**; …; Never do
McEwen & West (2001) UK [40] ****	N = 303, GPs, RR: 61	Male: 68, Age: Majority (40 between 40–49)	Self-report questionnaire, Distribution: via post, Developed by the researchers, pilot-tested, 41 items	n.r.	57, "Do you check their smoking status and record changes whenever smokers attend the surgery?"	50, "Do you advise smokers to stop during most or all consultations?"	n.r.	83, either recommended or prescribed NRT	n.r.	**Yes**; No
McEwen et al. (2005) UK [41] ****	N = 336, GPs, RR: 63	Male: 57, Age: Majority (40 between 40–49)	Self-report questionnaire, Distribution: via post, Developed by the researchers based on previous questionnaires, 33 items	n.r.	63, "Do you check their smoking status and record changes whenever smokers attend the surgery?"	51, "Do you advise smokers to stop during most or all consultations?"	n.r.	70, refer patients to their local specialist smoking cessation service	n.r.	**Yes**; No
McLeod et al. (2000) New Zealand [42] New ***	N = 283, GPs, RR: 63	n.r.	Self-report questionnaire, Distribution: via post, fax	n.r.	80, Extent to which patients were asked if they smoked	n.r.	n.r.	n.r.	16, Extent to which a patient's progress in smoking cessation was followed up	n.r.
Meshefedjian et al. (2010) Canada [43] ***	N = 610, GPs, RR: 70	Male: 55, Age: Majority (52 between 40–54)	Self-report questionnaire, Distribution: via post, telephone	n.r.	91, Ascertains smoking status of patients	76, Provides advice on how to quit	n.r.	26, Provides adjunct support (offered written educational materials, follow-up visits, etc.)	n.r.	**(All**; **Almost all**; **More than half);**(About half; Less than half; Few/none)
Ng et al. (2007) Indonesia [44] ***	N = 447, PCPs, RR: 65	Male: 53, Age: n.r.	Self-report questionnaire, Distribution: in person, Developed by the researchers based on previous questionnaires	n.r.	Fem.: 34, Male: 21, "During a consultation, do you ask patients whether they smoke?"	n.r.	n.r.	n.r.	n.r.	Always; almost always; occassionally; never
Nobile et al. (2014) Italy [45] ****	N = 722, PCPs, RR: 69	Male: 68.3, Age: majority (54) between 51–59	Self-report questionnaire, Distribution: via internet	n.r.	89, [Ask all patients if they smoke]	99, Recommend all smokers to quit smoking	26, Classify smoking patients in relation to their motivation to quit	86, Provide low intensity counseling of 3–10 Minutes to patients without other risk factors	n.r.	n.r.
O'Loughlin et al. (2001) Canada [46] ***	N = 337, GPs, RR: 77	Male: 61, Age: *M* = 45.3 ± 9.5	Self-report questionnaire, Distribution: via post, 89 items	n.r.	Fem.: 56, Male: 43	84, "For which patients who did not want to quit did you express concern about patient's continued smoking?"	62, "For which smoking patients who where preparing to quit did you discuss setting a quit date?"	75, "For which smoking patients who where preparing to quit did you recommend NRT?"	50, "For which smoking patients who where preparing to quit did you offer a follow-up visit 1 or 2 weeks after cessation?"	Ask: **Always; Usually; Sometimes;** Rarely; Never, other A's: **All patients**; **almost all**; **more than half**; about half; less than half; few/no
Pipe et al. (2009) France, Germany, Greece, Italy, Japan, Korea, Mexico, Netherlands, Poland, Spain, Sweden, Switzerland, Turkey, UK, US [47] ***	N = 2836, GPs, FPs, RR: 63	Male: 76, Age: smokers: *M* = 48, non-smokers: *M* = 49	Self-report questionnaire, Distribution: via telephone, face-to-face interviews, Developed by the researchers, pilot-tested	Potential consultation activities were read from a list and physicians were asked to identify the activities they had completed during their most recent consultation	Smokers: 80 Non-smokers: 85, Ask how much the patient smokes	Smokers: 85, Non-smokers: 90, Advise the patient to quit smoking	n.r.	Smokers: 40, Non-smokers: 49, Assist the patient to develop a plan to quit	n.r.	**Yes**; No
Schneider et al. (2014) Germany [48] ****	N = 4074, GPs, internists, RR: n.r.	Male: 60, Age: *M* = 51 ± 9	Self-report questionnaire, Distribution: via post, Developed by experts, pilot-tested	n.r.	90, Assessed tobacco consumption	82, Advised smokers to stop smoking	11, Agree on a goal	76, Assisted in the form of a brief intervention	54, Arranged a follow-up appointment	n.r.
Schnoll et al. (2006) US [49] ****	N = 1120, GPs, FPs, internists, RR: 62	Male: 79, Age: *M* = 49 ± 11.1	Self-report questionnaire, Distribution: via post, Developed by interviews/focus groups with experts, literature review	n.r.	n.r.	75, "Among patients you have identified as smokers, how often do you advise them to stop smoking?"	n.r.	13, "For your patients who want to quit smoking, how often do you recommend nicotine patch?"	n.r.	**Always**; often; sometimes; never
Skoeries et al. (2010) Germany [50] **	N = 637 GPs, RR: 54	Male: 41.3, Age: *M* = 53	Self-report questionnaire, Distribution: via post	n.r.	30, Document the smoking status during the first consultation	13, Offer advice to all of their smoking patients	n.r.	n.r.	n.r.	**Always**; often; sometives; rarely; never
Soto Mas et al. (2005) US [51] *	N = 45, PCPs, RR: 56	Male: 73.3, Age: Majority (48.9) between 36–45	Self-report questionnaire, Distribution: via post, Developed using qualitative and quantitative methods, pilot-tested, 50 items	n.r.	44, Ask patients about smoking status	42, Advise smoking patients to quit	n.r.	25, Assist smoking patients by talking to them about the health risks of smoking, providing materials or referring	4, Arrange follow-up for smoking patients-follow-up visits or phone calls	**>80% of patients**; 61–80%; 41–60%; 20–40%; <20%
Squier et al. (2006) Ukraine [52] **	N = 799, GPs, RR: n.r.	Male: 35.9, Age: *M* = 45	Self-report questionnaire, Distribution: in person, Developed based on existing literature, 70 items	n.r.	98, Asked their patients about tobacco use during the past month	n.r.	n.r.	n.r.	n.r.	**Asks or knows and records**; **Does not record**; Does not know
Tanriover et al. (2014) Turkey [53] ***	N = 164, FPs, RR: 82	Male: 47.6, Age: *M* = 34.8 ± 8.4	Self-report questionnaire, Distribution: n.r., Developed by literature review and consensus between researchers, pilot-tested, 50 items	n.r.	79, "In my history taking I always ask about the current smoking status of the patient."	n.r.	n.r.	n.r.	n.r.	**Yes**; No
Ulbricht et al. (2006) Germany [9] ***	N = 37, GPs, RR: 87	Male: 51.2, Age: *M* = 47.5 ± 8.24	Self-report questionnaire, Distribution: in person		7, "Do you routinely assess the smoking status when a patient attends the practice?"	n.r.	n.r.	n.r.	n.r.	**Regularly**; occasionally; rarely or never
Varona et al. (2005) Cuba [54] ***	N = 114, FPs, RR: n.r.	Male: 33, Age: n.r.	Self-report questionnaire, Distribution: in person, Developed by the researchers, pilot-tested	n.r.	n.r.	35, Recommended smoking cessation	32, Asked their patients if they intended to stop smoking	38, Gave advice on how to achieve smoking cessation	n.r.	n.r.
Young & Ward (2001) Australia [55] ***	N = 311, GPs, RR: 73	Male: 69, Age: *M* = 45	Self-report questionnaire, Distribution: via post, Developed by the researchers, pilot-tested	n.r.	34, Provide smoking cessation advice at every patient visit	32, Advise patients to quit completely	72, Always or frequently assess patient's stage of change	28, Negotiate a quit date	n.r.	Ask:**Almost every visit**; at intervals or when symptomatic; initial visit only; Never, other A's: **Always**; **frequently**; sometimes; occasionally; never

*Legend*. More asterisks indicate a higher quality grade according to the Mixed Methods Appraisal Tool (MMAT; * Met 25% of MMAT criteria; ** Met 50% of MMAT criteria; *** Met 75% of MMAT criteria; **** Met 100% of MMAT criteria), see S4 File for further information; GP = General practitioner; FP = Family practitioner; N = Sample size; *M* = Mean; n.r. = not reported.

^a^ Questions in quotation marks are reported in their exact wording; and all other questions are reported semantically.

^b^ Response options in bold correspond to the percentages reported for the 5 A's.

### Data synthesis

The main outcome was the proportion of physicians reporting counseling and in cases where multiple items were used to measure smoking cessation counseling behavior, the item that best represented the respective 5A’s strategy was chosen. In the case of rating scales, we reported the responses to the end point of the scale. For example, for the response options “Never”, “Occasionally”, “Almost always” and “Always”, the proportions of physicians that selected “Always” were reported. If multiple forms of assistance were reported (e.g. prescribing NRT, handing out leaflets, referral to expert), we reported only the most frequently offered form of assistance.

## Results

### Study selection

**Fig 1:**
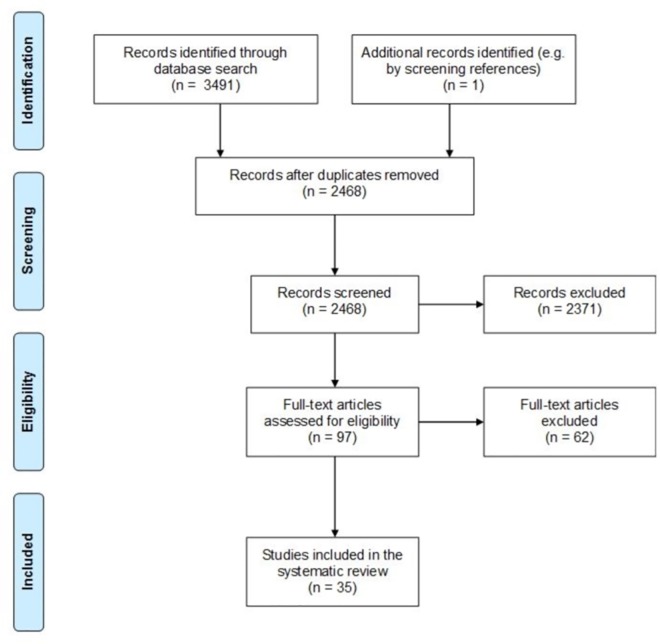
Flow Diagram.

Overall, 3491 records were identified by the electronic database search. After duplicates were removed, the title and abstracts of 2468 articles were screened according to inclusion/exclusion criteria. The remaining 97 full-text articles were assessed for eligibility. Of these articles, 62 were excluded (see S3 File for a list of the reasons for exclusion). The remaining 35 articles were included in the final review. The process for including/excluding studies is illustrated in the flow diagram in Fig 1 [16].

### Description of included articles

In total, 35 articles were included in the review and reported studies from 17 countries (see Table 2 for details). Sample sizes from studies included in the review ranged from 37 to 4074 (mean = 809). Thirty-one studies reported response rates, with a mean response rate of 64% (range: 18% to 95%). Thirty-four studies used survey designs and 1 article reported an intervention study (single group, pre-post design) [9] of which baseline data were included. Surveys were mainly distributed via post (16 studies) or in person (7 studies). Descriptive data of all studies included in the review are shown in Table 2.

### Smoking cessation counseling

Of the 35 articles, 8 articles reported counseling behavior corresponding to all 5 counseling strategies (ask, advise, assess, assist and arrange) [7, 23, 26, 29, 31, 32, 36, 46]. The strategies “Ask” and “Advise” were most frequently reported. “Ask” was reported in 29 articles and on average 65% (range: 7% to 100%) of physicians asked about their patient’s smoking behavior. Twenty-five articles reported “Advise” and on average, 63% of physicians (range: 13% to 99%) advised their patients to stop smoking. Fourteen articles reported behavior corresponding to “Assess” with an average of 36% (range: 11% to 72%) of physicians assessing their patient’s smoking status. Twenty-three articles reported “Assist”, with an average of 44% (range: 2% to 98%) of physicians providing assistance. Fourteen articles reported “Arrange” and on average 22% (range: 2% to 54%) of physicians arranged follow-up consultations. Table 2 shows the proportions of physicians offering smoking cessation counseling, categorized according to the 5A’s.

The items and response options used in each study are shown in Table 2. Whereas some researchers used a single item to measure counseling behavior, others used multiple items. For example, counseling behavior corresponding to the “Ask” strategy was assessed with the item "During a consultation, do you ask patients whether they smoke?" in one study [44], whereas another study [35] used the items "I ask patients at their annual visits if they use tobacco", "When patients come in for unrelated problems, I ask them about tobacco use" to measure behavior. When multiple items were used to measure counseling behavior in a single article, the responses to the item that most accurately represented the strategy of interest was reported. For example, the items ‘‘Do you assess the smoking status when a patient attends the practice for the first time?” and “Do you routinely assess the smoking status when a patient attends the practice?” [9] both concern asking patients about tobacco use and therefore relate to the strategy “Ask” (systematically identify all tobacco users at every visit). However, the second item corresponds to the strategy more closely because it concerns asking *all* patients about tobacco use (not only upon the first visit to the practice office). Therefore, the responses to the second item are reported in the review.

The questionnaires used also differed between studies and some researchers used adapted versions of existing questionnaires to measure counseling behavior. For example, one study used a 45-item questionnaire that was based on a questionnaire by the World Health Organization [28, 56]. In contrast, other studies included questionnaires developed by the researchers during project group discussions or expert consultations (e.g. in-depth interviews). Further differences were seen in the response options of questionnaire items. Dichotomous response scales (“Do you ask patients whether he or she smokes?”; “yes”,”no”) [37] were used in some studies, whereas others used rating scales (“During a consultation, do you ask patients whether they smoke?”; “never”, “occasionally”, “almost always” and “always”) [44]. When a rating scale was used to assess counseling behavior, only data from the end point of the scale was reported. For example, in order to extract counseling behavior for the strategy “Ask”, we reported the proportion of physicians who responded “always” to the item “During a consultation, do you ask patients whether they smoke?”

### Quality of included articles

The quality assessment is included in Table 2. Details of the quality assessment are shown in S4 File. There was moderate agreement between the two raters on the assessment of study quality (Cohen’s Kappa = 0.59). Any discrepancies were resolved by discussion. The methodological quality of the included studies varied and the population sizes were between N = 37 to N = 4074. Also, information on the instruments used to measure physicians’ smoking cessation counseling was often insufficient or lacking and the information available is displayed in Table 2. However, no study was excluded due to its methodological quality.

## Discussion

The aim of this review was to systematically assess the frequency of behavioral counseling for smoking cessation in primary care and the 5A’s strategy was used to structure smoking cessation counseling. Main findings are that the first strategies “Ask” and “Advise” were more often reported than the subsequent strategies “Assess”, “Assist” and “Arrange”. This finding corresponds to research that found the last two strategies (assist, arrange) to be least reported [57]. However, it is not possible to say whether individual steps were not performed by physicians or simply not measured or reported by the researchers. Secondary findings are that the proportions of physicians reported differed considerably per 5A strategy. For example, 81% (95% CI = 74%, 88%) of physicians reported to give advice in one study [29], whereas only 13% (10%, 16%) of physicians gave advice according to another study [50]. While differences in proportions may result from variations in sample characteristics and settings, they may also be due to the wording of questionnaire items used to assess counseling behavior [13]. More physicians may have indicated advising patients in the first study [29] because the item (“Do you advise the patient to quit smoking?”) can be understood to refer to only some or all patients. In contrast, physicians in the second study [50] were specifically asked if they offered advice to all of their patients. These differences could also explain why fewer physicians indicated giving advice in the second study.

Also, there are different forms of assistance, such as providing supporting information, referring to quitlines or recommending the use of pharmacotherapy. According to a literature review, assistance was most frequently offered in form of educational materials [13]. In contrast, the results of the present review suggest that nicotine replacement therapy (NRT) was the most frequently offered form of assistance. For example, one study reported that 61% (57%, 65%) of physicians recommended NRT, whereas only 25% (22%, 28%) provided self-help materials [25]. However, it is difficult to clearly determine which form of assistance was offered most frequently because not all articles included in this review distinguished between different assistance types.

### Limitations

Risk of bias is seen in the self-report questionnaires used to measure physicians’ smoking cessation counseling activities. Self-report measures may introduce social desirability or recall bias [58]. In particular, physicians may overestimate their adherence to guidelines and the frequency with which they deliver preventive services [13, 17]. Therefore, a limitation is that studies presenting physician-reported but not patient-reported data were included in this review [17]. Besides, it has been proposed that instruments to assess the delivery of preventive services should take into account the perspective of both, physicians and patients [59, 60]. Also, physicians who participate in surveys on smoking cessation counseling may be more interested and hence more engaged in counseling activities than the overall population of physicians. Nevertheless, self-report measures make it possible to measure many participants and are therefore an appropriate research tool for this population [61]. While other methods such as video-based observation can be used to conduct research in primary care, these methods complicate the research process due to technical requirements and confidentiality and privacy aspects [62].

Moreover, differences in the items used to assess counseling behavior limited the comparability of study results. A systematic review on behavioral counseling for cardiovascular disease found that physician-reported smoking cessation counseling differed, depending on the phrasing of survey questions [13]. Moreover, the number of items and type of response option (dichotomous or rating scale) may influence reports of counseling behavior [43]. Further, it should be noted that the 5A’s strategy used to structure smoking cessation counseling was initially presented by the US public health service and may be less familiar outside of the US [5]. Although the strategy is mentioned in the guidelines of other countries, such as Canada [63], and Germany [64], specific recommendations may differ between countries. We still chose the 5A’s strategy because it distinguishes between 5 counseling steps and is therefore more comprehensive than other frameworks such as the ABC model [4, 5, 11]. Also, only articles in English and German language were included in the review, which means that relevant studies published in other languages may have been missed.

### Implications

While this review summarizes physicians’ perspective of smoking cessation counseling in practice, it does not provide information on the reasons why physicians do or do not offer counseling. Changes in health care policy and clinical guidelines affect provider behaviour and may influence the frequency and type of smoking cessation counseling. According to previous studies, self-efficacy beliefs, prior training and patient characteristics determine whether physicians offer counseling [13, 65]. For example, physicians may not approach patients who appear unmotivated or do not intend to quit smoking due to fear of harming the physician-patient relationship [66]. A systematic literature on the barriers to discussing smoking cessation with patients exists [66] and future studies could explore a possible association between attitudes towards counseling and actual counseling behavior [13]. This would help to further understand and address common barriers. Also, future studies could include patient-reported or more objective data, in order to gain further insights into the implementation of smoking cessation counseling in practice.

In concluding, more research is needed in order to monitor changes and draw firm conclusions about provider behavior. The present results suggest that there is need for a standardized instrument to assess counseling behavior that differentiates between the possible forms of advice and assistance (e.g. NRT, referral to specialist) because this would help to identify current gaps in practice. While instruments to assess smoking cessation counseling have been developed in relation to the 5A’s strategy, these instruments require additional evaluation. Most instruments remain unpublished and data on reliability and validity are often missing [57].

## Supporting Information

S1 File Database search. (PDF)

S2 File PRISMA Checklist. (PDF)

S3 File Full-text articles excluded by reason. (PDF)

S4 File Details of MMAT-Criteria. (PDF)
