# Supplementary material for: A Systematic Literature Review of Self-Reported Smoking Cessation Counseling by Primary Care Physicians
Source: PLoS One. 2016 Dec 21;11(12):e0168482. doi: 10.1371/journal.pone.0168482 (PMC5176294; doi:10.1371/journal.pone.0168482)
Supplement: S1 File — (PDF) [file pone.0168482.s001.pdf]

S1 File. Database search.

Database search overview

Database	Date of search	Time frame/update status	Hits
Embase (OvidSP)	10.7.2015	1980 until 2015 Week 27	1928
Medline (OvidSP)	10.7.2015	1946 until June Week 4 2015	661
PsycINFO (OvidSP)	10.7.2015	1806 until June Week 5 2015	235
CINAHL	10.7.2015		232
Cochrane Library	10.7.2015		435
		**Total with duplicates**	3491
		**Total without duplicates**	2467

EMBASE via OVID on 10th July 2015

Nr.	Search terms	Hits
1	exp smoking cessation/ or exp smoking cessation program/ or exp nicotine replacement therapy/	42929
2	((smoking or nicotine or tobacco) and (stop$ or cessation or quit$ or anti)).mp.	66777
3	exp counseling/ or exp patient referral/	181384
4	(ask or advi?e or assess or assist$ or arrange or refer$ or consult or recommend).mp.	2623235
5	exp general practice/ or exp general practitioner/ or exp primary medical care/	180782
6	(family physician? or primary care physician? or general practitioner? or practitioner?).mp.	237029
7	(family practice or family medicine).mp.	19291
8	1 or 2	67177
9	3 or 4	2711250
10	5 or 6 or 7	334621
11	8 and 9 and 10	2284
12	limit 11 to yr="2000-2015"	1952
13	remove duplicates from 12	1928

Medline via OVID on 10th July 2015

Nr.	Search terms	Hits
1	exp smoking cessation/ or exp smoking cessation program/ or exp nicotine replacement therapy/	21856
2	((smoking or nicotine or tobacco) and (stop$ or cessation or quit$ or anti)).mp.	43852
3	exp counseling/ or exp patient referral/	34865

4	(ask or advi?e or assess or assist$ or arrange or refer$ or consult or recommend).mp.	1760712
5	exp general practice/ or exp general practitioner/ or exp primary medical care/	67949
6	(family physician? or primary care physician? or general practitioner? or practitioner?).mp.	129887
7	(family practice or family medicine).mp.	65161
8	1 or 2	43852
9	3 or 4	1787364
10	5 or 6 or 7	176981
11	8 and 9 and 10	953
12	limit 11 to yr="2000-2015"	682
13	remove duplicates from 12	661

PsycINFO via OVID on 10th July 2015

Nr.	Search terms	Hits
1	exp smoking cessation/ or exp smoking cessation program/ or exp nicotine replacement therapy/	10004
2	((smoking or nicotine or tobacco) and (stop$ or cessation or quit$ or anti)).mp.	15572
3	exp counseling/ or exp patient referral/	68797
4	(ask or advi?e or assess or assist$ or arrange or refer$ or consult or recommend).mp.	456188
5	exp general practice/ or exp general practitioner/ or exp primary medical care/	5011
6	(family physician? or primary care physician? or general practitioner? or practitioner?).mp.	74320
7	(family practice or family medicine).mp.	3314
8	1 or 2	15572
9	3 or 4	513578
10	5 or 6 or 7	76338
11	8 and 9 and 10	286
12	limit 11 to yr="2000-2015"	235
13	remove duplicates from 12	235

CINAHL on 10th July 2015

Nr.	Search terms	Hits
1	(MH "Smoking Cessation") OR (MH "Smoking Cessation Programs") OR (MH "Tobacco Use Cessation Products")	11769
2	((smoking or nicotine or tobacco) and (stop* or cessation or quit* or anti))	15373
3	(MH "Counseling+")	17987
4	(ask or advi?e or assess or assist* or arrange or refer* or consult or recommend)	291209

5	(MH "Primary Health Care")	31256
6	(MH "Physicians, Family")	8467
7	(MH "Family Practice")	10773
8	S1 OR S2	15309
9	S3 OR S4	306000
10	S5 OR S6 OR S7	45875
11	S8 AND S9 AND S10	262
12	limit 11 to yr="2000-2015"	232

The Cochrane Library on 10th July 2015

Nr.	Search terms	Hits
1	MeSH descriptor: [Tobacco Use Cessation] explode all trees	2940
2	(("smoking" or "nicotine" or "tobacco") and ("stop*" or "cessation" or "quit*" or "anti"))	8473
3	MeSH descriptor: [Counseling] explode all trees	3455
4	("ask" or "advi?e" or "assess" or "assist*" or "arrange" or "refer*" or "consult" or "recommend")	140903
5	MeSH descriptor: [Physicians, Primary Care] explode all trees	71
6	("family physician?" or "primary care physician?" or "general practitioner?" or "practitioner?")	7282
7	("family practice" or "family medicine")	4924
8	#1 or #2	8473
9	#3 or #4	143056
10	#5 or #6 or #7	10964
11	#8 and #9 and #10 Publication year from 2000 to 2015	435
