# Supplementary material for: A Systematic Literature Review of Self-Reported Smoking Cessation Counseling by Primary Care Physicians
Source: PLoS One. 2016 Dec 21;11(12):e0168482. doi: 10.1371/journal.pone.0168482 (PMC5176294; doi:10.1371/journal.pone.0168482)
Supplement: S3 File — (PDF) [file pone.0168482.s003.pdf]

# S3 File. Full-text articles excluded by reason.

| | Exclusion criteria | Excluded full texts (n = 62) |
|---|---|---|
| **A.** | Study population are not primary care physicians (defined as general practitioners, family physicians or internists). | 3 |
| **B.** | Study does not report on smoking cessation counseling delivered by primary care physicians to patients. Patients receiving the intervention are below age 18. | 0 |
| **C.** | Study does not report proportion of primary care physicians | 0 |
| **D.** | Study does not report proportions of primary care physicians engaging in smoking cessation counseling. | 39 |
| **E.1** | Measure is not a self-report questionnaire. | 5 |
| **E.2** | Intervention study and no baseline data reported. | 3 |
| **E.3** | Not published in English or German language. | 11 |
| **E.4** | Article is a dissertation. | 0 |
| **E.5** | Article is a conference proceeding. | 0 |
| **E.6** | Article is a qualitative study. | 0 |
| **E.7** | Article is an unpublished manuscript. | 0 |
| **E.8** | Full text not available. | 1 |
