# Supplementary material for: A Systematic Literature Review of Self-Reported Smoking Cessation Counseling by Primary Care Physicians
Source: PLoS One. 2016 Dec 21;11(12):e0168482. doi: 10.1371/journal.pone.0168482 (PMC5176294; doi:10.1371/journal.pone.0168482)
Supplement: S4 File — (PDF) [file pone.0168482.s004.pdf]

S4 File. Details of MMAT-Criteria.

#	Criteria	Studies and scores																																		
**A**	**Screening questions**	**1**	**2**	**3**	**4**	**5**	**6**	**7**	**8**	**9**	**10**	**11**	**12**	**13**	**14**	**15**	**16**	**17**	**18**	**19**	**20**	**21**	**22**	**23**	**24**	**25**	**26**	**27**	**28**	**29**	**30**	**31**	**32**	**33**	**34**	**35**
1	Clear qualitative/quantitative research or mixed method question?	✓ *	✓ *	✓ *	✓ *	✓ *	✓ *	✓ *	✓ *	✓ *	✓ *	✓ *	✓ *	✓ *	✓ *	✓ *	✓ *	✓ *	✓ *	✓ *	✓ *	✓ *	✓ *	✓ *	✓ *	✓ *	✓ *	✓ *	✓ *	✓ *	✓ *	✓ *	✓ *	✓ *	✓ *	✓ *
2	Do the collected data allow addressing the research question?	✓ *	✓ *	✓	✓ *	✓ *	✓ *	✓ *	✓ *	✓ *	✓ *	✓ *	✓ *	✓ *	✓ *	✓ *	✓ *	✓ *	✓ *	✓ *	✓ *	✓ *	✓ *	✓ *	✓ *	✓ *	✓ *	✓ *	✓ *	✓ *	✓ *	✓ *	✓ *	✓ *	✓ *	✓ *
**C**	**Quantitative descriptive**	**1**	**2**	**3**	**4**	**5**	**6**	**7**	**8**	**9**	**10**	**11**	**12**	**13**	**14**	**15**	**16**	**17**	**18**	**19**	**20**	**21**	**22**	**23**	**24**	**25**	**26**	**27**	**28**	**29**	**30**	**31**	**32**	**33**	**34**	**35**
1	Is the sampling strategy relevant to address the quantitative research question?	✓ *	✓ *	✓ *	✓ *	✓ *	✓ *	✓ *	✓ *	✓ *	✓ *	✓ *	✓ *	✓ *	✓ *	✓ *	✓ *	✓ *	✓ *	✓ *	✓ *	✓ *	✓ *	✓ *	✓ *	✓ *	✓ *	✓ *	✓ *	✓ *	✓ *	✓ *	✓ *	✓ *	✓ *	✓ *
2	Is the sample representative of the population understudy?	✓ *	✓ *	✓ *	✓	✓	✓ *	✓	✓	✓ *	✓	✓	✓	✓ *	✓	✓ *	✓ *	✓	✓ *	✓ *	✓ *	✓ *	✓	✓	✓ *	✓	✓	✓ *	✓ *	✓	✓	✓	✓	✓	✓ *	✓
3	Are measurements appropriate?	✓ *	✓ *	✓ *	✓ *	X	✓ *	✓ *	✓ *	✓ *	✓ *	✓ *	✓ *	✓ *	✓ *	✓ *	✓ *	✓ *	✓ *	✓ *	✓ *	X	✓ *	✓ *	✓ *	✓ *	✓ *	✓ *	✓ *	✓ *	X	✓ *	✓ *	✓ *	✓ *	✓ *
4	Is there an acceptable response rate (60% or above)?	✓	✓ *	✓ *	✓	X	✓	✓ *	✓ *	✓ *	✓	✓ *	✓ *	✓ *	✓	✓ *	✓ *	✓ *	✓	✓ *	✓ *	✓ *	✓ *	✓ *	✓ *	✓ *	✓ *	✓ *	✓ *	✓	✓	X	✓ *	✓ *	X	✓ *
**E**	**Total score**	*******	********	********	******	*****	*******	*******	*******	********	**	*******	*******	********	******	********	********	*******	*******	********	********	*******	*******	*******	********	*******	*******	********	********	******	*****	******	*******	*******	*******	*******

1[19], 2[20], 3[21], 4[22], 5[17], 6[23], 7[24], 8[25], 9[26], 10[27], 11[28], 12[29], 13[30], 14[31], 15[32], 16[33], 17[34], 18[35], 19[32], 20[37], 21[38], 22[39], 23[40], 24[41], 25[42], 26[43], 27[44],28[45], 29[46], 30[47], 31[48], 32[49], 33[7], 34[50], 35[51] x MMAT criteria not described; ✓ MMAT criteria described / = Met 0% of MMAT criteria; * Met 25% of MMAT criteria; ** Met 50% of MMAT criteria; *** Met 75% of MMAT criteria; **** Met 100% of MMAT criteria
